# The effects of water immersion and epidural analgesia on cellular immune response, neuroendocrine, and oxidative markers

**DOI:** 10.3906/sag-2009-181

**Published:** 2021-06-28

**Authors:** Özlem UZUNLAR, Ümit Yasemin SERT, Nezaket KADIOĞLU, Tuba ÇANDAR, Yaprak ENGİN ÜSTÜN

**Affiliations:** 1 Department of Obstetrics and Gynecology, Ankara City Hospital, University of Health Science, Ankara Turkey; 2 Department of Obstetrics and Gynecology, Liv Hospital, Ankara Turkey; 3 Department of Biochemistry, Ufuk University, Ankara Turkey; 4 Department of Obstetrics and Gynecology, Etlik Zubeyde Hanım Education and Research Hospital, University of Health Science, Ankara Turkey

**Keywords:** Epidural analgesia, infection, neuroendocrine, oxidative stress, waterbirth

## Abstract

**Background/aim:**

Water immersion and epidural analgesia are the most preferred pain relief methods during the labor process. Adverse effects related to these methods, impact on the labor, and perception of pain is well studied in the literature. We aimed to investigate the cord blood level of copeptin, total serum oxidant (TOS), antioxidant (TAS), interleukin (IL)-1, IL-6, and oxytocin after the labor with water immersion, epidural analgesia, and vaginal birth without pain relief.

**Materials and methods:**

The study was conducted with 102 healthy pregnant women admitted to the obstetric delivery unit for noncomplicated term birth. Copeptin, oxytocin, TAS, TOS, IL-1, and IL-6 levels of cord blood and obstetric and neonatal results after vaginal birth were compared.

**Results:**

The study included a total of 102 patients (group 1 = 30, group 2 = 30, and group 3 = 42). We found no significant difference between the three groups in terms of BMI, age, gravidity, parity, birth week, birth weight, interventional birth, perineal trauma, breastfeeding, duration of labor, oxytocin, IL-1 and IL-6 levels (p > 0.05). Neonatal intensive care unit (NICU) need, TAS, TOS, and copeptin levels were higher. Apgar scores were lower in the epidural group (p = 0.011, p = 0.036, p = 0.027, p < 0.001, and p < 0.001 respectively).

**Conclusion:**

Epidural analgesia has deteriorated oxidative stress status and lower neonatal Apgar scores with higher NICU administration compared with water birth and vaginal birth without pain relief.

## 1. Introduction

The International Association for the Study of Pain describes pain as an unpleasant sense and emotion related to real or potential tissue damage [1]. The pain of labor is known as a pain that is very intense and difficult to bear, although it is subjective and affected by several physiological and psychological factors [2]. The labor pain is associated with a stretch of the uterus, cervix, vagina, and distension of pelvic ligaments and muscles [2]. Several pharmacologic and nonpharmacologic pain relief methods were described in the literature to decrease the unpleasant feeling and increase maternal satisfaction with a positive experience and compliance [2]. Water immersion is one of the most studied and debated nonpharmacological methods, while epidural analgesia is a frequently used pharmacological method nowadays. Both ways are primarily evaluated regarding maternal and fetal concerns in the literature.

Copeptin is a glycopeptide stored in neuro-hypophyseal vesicles with arginine vasopressin (AVP) and neurophysin II [3]. Myocardial infarction, sepsis, heart failure, stroke, diabetes insipidus, hyponatremia, and metabolic diseases are the main fields in which the copeptin levels are used [3]. It is known that copeptin increases during stressful conditions, and it is believed to be a more reliable marker than cortisol to reflect the stress level of the body [3]. The level of copeptin during labor is a sensitive stress marker of the birth process [4].

Oxytocin is a peptide hormone produced from the neurons of the paraventricular and supraoptic nuclei of the hypothalamus [5]. The birth process needs oxytocin to create uterine contractions and prepare the women for the birth psychologically [6]. The plasma level of oxytocin changes during the pregnancy and increases by frequency and amplitude with progressing labor [5]. Uterine muscles become more sensitive to oxytocin due to the up-regulation of receptors at the end of the pregnancy and during labor [7]. Oxytocin’s central effect includes amnesia, the opiate-like effect for less pain experience, positive emotions, and healthy interaction between mother and fetus [5].

Total serum oxidant (TOS) and total antioxidant (TAS) levels are essential markers reflecting the oxidative balance [8]. Reperfusion of ischemic tissue is associated with a wide and complex series of inflammatory reactions, which leads to tissue damage during the induction of oxidative stress [9,10]. Oxidative stress and inflammation-mediated situations were evaluated together in different subjects in the literature by measuring TAS, TOS, and cytokines together [11]. During normal labor, uterine contractions lead to ischemia in the uterine bloodstream followed by reperfusion [12]. In addition to the oxidative balance and inflammatory cytokines are deteriorated more seriously by severe adverse pregnancy outcomes [13,14].

This prospective study aimed to evaluate the cord level of copeptin, TAS, TOS, IL-1, IL-6, and oxytocin of vaginal births, after the labor with water immersion, epidural analgesia, and vaginal birth without pain relief. 

## 2. Materials and methods

**Figure 1:**
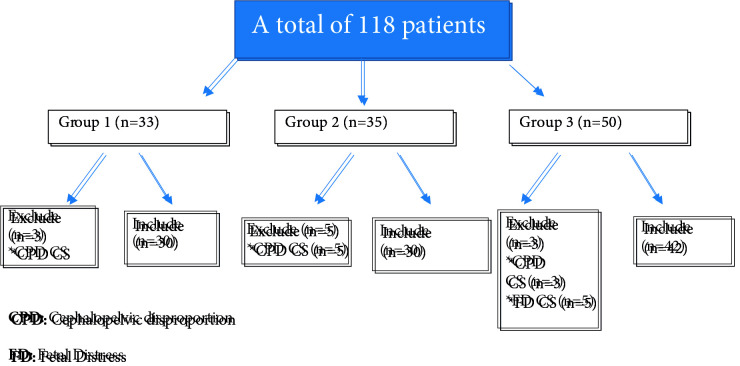
The patients who were excluded and included in the study and reasons for excluding.

The study is planned as a prospective study, including three groups. The study was performed between December 2018 and December 2019. Group 1 consisted of the term pregnant women who were submerged during the first stage of labor; group 2 included the pregnant women who had epidural analgesia for labor pain relief; group 3 involved the pregnant women who requested no pain relief method. All the women had a vaginal birth. The ethical committee approved the study of our hospital (38/2018). All the women gave written informed consent to be a part of this clinical research before the study. The study was planned with 118 patients including 33 women in group 1, 35 women in group 2, and 50 women in group 3. Inclusion criteria for the study were termed as, Turkish pregnant women without obstetrical risk factors. Known physical or psychological illness, an obstetrical complication such as diabetes, hypertension, fetal anomaly, fetal growth restriction, multifetal pregnancies, macrosomia, fetal distress, previous uterine surgery, malpresentation, any kind of infection existence or suspicion, membrane rupture for more than 6 h, in vitro fertilization pregnancies, active smoking during the pregnancy, and known chronic disease and medication were excluded from the study. The patients who have gone to the cesarean section were also excluded. Three patients from group 1 (3 cephalopelvic disproportion (CPD)), 5 patients from group 2 (5 CPD), and 8 patients from group 3 (5 fetal distress and 3 CPD) were excluded from the study because of going to the cesarean operation. The study included 102 patients. Groups 1, 2, and 3 included 30, 30, and 42 pregnant women, respectively. Figure 1 shows the patients who were excluded and included in the study and reasons for excluding.

### 2.1. Variables, data sources, and collection

Age, gravidity, parity, body mass index (BMI), gestational week, birth weight, interventional delivery, perineal trauma, duration of labor, and breastfeeding status 30 min after the delivery were collected from medical records.

Water immersion was offered to the women at the first stage of labor when the labor is active. Active labor was considered as cervical ripening more than 5 cm and intense regular contractions. No exogenous oxytocin administration was used for the patients included in the study. A standard immersion tube was used at the temperature of 34–36 °C, and women were taken to land at the second phase of labor. The fetal heart rate and vital signs of the women were controlled at regular intervals (every 30 min). The standard immersion contained being in the water during the entire labor. Activities such as leg movements and squatting down were carried out in the water. It was permissible to have a 5 min break for walking or going to the bathroom if the delivery was not too soon. The vaginal examination was performed at least once an hour to assess the labor progress. The patient was placed on the labor table when the fetal head crowning. Labor was performed on the ground?.

Epidural analgesia was offered at the active stage (between 3–7 cm) of labor to prevent labor interruption. The baseline pain was evaluated with a visual analogue scale (VAS) and the results of more than five were accepted appropriate for epidural analgesia. All the patients were hydrated with 10 mL/kg ringer’s lactate solution before the procedure for 30-min. The procedure was performed by tilting the patient forward or lateral decubitus. The iliac crest was used as a landmark for the fourth lumbar vertebra to determine the termination of the spinal cord. The level of epidural analgesia was between L2-L3 or L3-L4 vertebra to provide analgesia from T10 to S2-4. The sites of epidural analgesia have been prepared with a tincture of iodine. A 16-gauge Touhy needle was used vertically through the axis of the spine. Epidural space was identified by using the loss of resistance technique. If there is no sign of intravascular or intrathecal injection, the catheter was placed about 3–4 cm into the epidural space. Patients were positioned with the left lateral side. The head of the patient was elevated and a test dose of 3 mL lidocaine (1%) was administrated. Maternal systolic, diastolic blood pressure, heart rate, and oxygen saturation were monitored for 5 min. Epidural analgesia was performed with 2 μg/mL fentanyl and 10 mL bupivacaine (0.125%). Repeat dose was administrated every one and a half hours according to the VAS scores of the patients with the same dose of fentanyl and bupivacaine. The VAS intensity score consisted of a 100-mm line between no pain end and worst pain end. Patients were asked to point their current pain on the VAS scale. VAS scale was categorized as slight pain (1–3), moderate pain (4–6), and heavy pain (7–10). VAS scores were calculated every one and a half hours to determine the actual pain intensity. It was thought to be remarkable to administer an additional dose when the VAS score exceeds five. Motor block, hypotension, weakness of lower extremities, nausea-vomiting, itching, and sedation were observed and appropriate treatments were applied. The fetal heart rate and vital signs of the women were controlled at regular intervals (every 30 min). The vaginal examination was performed to assess labor progress and commencement of labor. Epidural catheters were taken out after the delivery. 

Group 3 included the patients who had no pain relief method and no medications as uterotonics. Spontaneous labor has occurred. The fetal heart rate and vital signs of the women were controlled at regular intervals (every 30 min). The vaginal examination was performed every 1-h or more frequently to assess labor progress and commencement of labor.

After the vaginal birth, the cord was clamped, and 5–10 cm3 cord blood from the umbilical artery was transferred to the tubes to be centrifuged for ten min at 5000 rpm . The sera of centrifuged blood were stored at specialized fridges with –80 °C temperature until the assays were performed. 

**Assay for Copeptin:** Copeptin level was calculated by the CPP ELISA kit (Fine Test/Wuhan Fine Biotech Co., Ltd) at ELISA reader (branded MRC UT6100, UK). All the results were reported as pg/mL. Intra-assay and inter-assay coefficient variation (CV%) were < 8% and < 10% respectively.

**Assay for IL-1 and IL-6** **:** IL-1 and IL-6 level was calculated by using human interleukin 1 alpha (IL-1 alpha) and human interleukin 6 (IL-6) ELISA kit (Fine Test/Wuhan Fine Biotech Co., Ltd) at ELISA reader (branded MRC UT6100, UK). All the results were reported as pg/mL. Intra-assay and inter-assay coefficient variation (CV%) were < 8% and < 10% respectively.

**Assay for oxytocin** **:** Oxytocin level was calculated by OT (oxytocin) ELISA kit (Fine Test/Wuhan Fine Biotech Co., Ltd) at ELISA reader (branded MRC UT6100, UK). All the results were reported as pg/mL. Intra-assay and inter-assay coefficient variation (CV%) were < 8% and < 10% respectively.

**Assay for TAS and TOS:** Total oxidant status (TOS) was calculated spectrophotometric method by using the measurement kit (Rel Assay Diagnostic, Turkey). Measurements were made (Heales mb530 device, Shanghai, China), and all the results were reported as µmol/L. The normal range for human sera was reported as between 4.00 and 6.00 µmol/L. Intra-assay and inter-assay coefficient variation (CV%) were < 3.9% and < 3 .2% respectively.

Total antioxidant status (TAS) was measured spectrophotometric method by using the measurement kit of Rel Assay Diagnostic. Measurements were made with the Heales mb530 device, and all the results were reported as mmol/L. The normal range for human sera was reported as between 1.20 and 1.50 mmol/L. Intra-assay and inter-assay coefficient variation (CV%) were < 3.3% and < 2.8% respectively.

### 2.2. Statistical analysis

Data analysis was made using IBM SPSS Statistics v: 17.0 software (IBM Corp., Armonk, NY, USA). The Kolmogorov–Smirnov test determined the normal or not normal distributions of continuous variables. The Levene test was used to evaluate the assumption of homogeneity of variances. Descriptive statistics for continuous variables were determined as mean ± SD or median (25th–75th) percentiles. Simultaneously, the Kruskal–Wallis test calculated the continuous variables when parametrical test assumptions were not enough. One–way ANOVA compared the mean differences among groups. When the p-values by the Kruskal–Wallis test or One-way ANOVA were statistically significant, Dunn–Bonferroni or posthoc Tukey HSD test was used to differentiate the groups. Nominal data were determined by Pearson’s χ2 or Likelihood ratio test. Spearmen’s rho test was used to measure the strength of association between two variables. The value of r changed between 1 and –1 which means perfect positive correlation and perfect negative correlation respectively. A p-value of less than 0.05 was considered statistically significant.

## 3. Results

Demographic, maternal, and fetal characteristics regarding groups were presented in Table 1. We found no significant difference between the three groups in terms of age, body mass index (BMI), gravidity, parity, gestational week, and birth weight (p > 0.05). Duration of the first phase, duration of the second phase, delivery duration, interventional delivery, perineal trauma, and breastfeeding status was not statistically different between the three groups (p > 0.05).

**Table 1:** Demographical, maternal, and fetal characteristics regarding for groups.

	Group 1 (n = 30)	Group 2(n = 30)	Group 3 (n = 42)	p-value
Age (years)	27 (18–29)	29 (20–33)	29 (25–31)	0.510†
BMI (kg/m2)	29.2 (24–33.5)	28.6 (27.3–33.1)	29.0 (28.4–34.1)	0.453†
Gravidity	3 (1–4)	2 (1–3)	3 (2–4)	0.596†
Parity	2 (0–3)	1 (0–2)	1 (1–1)	0.794†
Gestational week	38.4 (38.0–41.0)	39.2 (38.0–39.5)	38.3 (38.0–40.5)	0.796†
Birth weight (g)	3250 (2800–3900)	3356 (3250–3710)	2950 (2875–3825)	0.653†
1st min APGAR	9 (8–9)a	8 (7–9)a,b	9 (8–9)b	<0.001†
5th min APGAR	10 (10–10)a	8.5 (8-9)a,b	10 (10–10)b	<0.001†
Interventional birth	15 (50.0%)	14 (46.6%)	22 (52.3%)	0.054‡
Need of neonatal intensive care unit	1 (3.3%)	6 (20%)	1 (2.3%)	0.011¶
Perineal trauma	2 (6.6%)	7 (23.3%)	2 (4.7%)	0.100¶
Breastfeeding*	21 (70.0%)	25 (83.3%)	23 (54.7%)	0.589‡
Duration of first stage (h)	9.0 (6.0–10.0)	9.0 (6.0–12.0)	10.0 (8.0–11.5)	0.552†
Duration of second stage (min)	40 (15–85)	43 (20–48)	37 (18–47)	0.608†

There was a statistically significant difference between the three groups in terms of 1st and 5th minutes APGAR scores. APGAR scores of group 2 at 1st and 5th minutes were significantly lower than group 1 and 3 (p < 0.001 and p < 0.001 respectively). There was no statistically significant difference between groups 1 and 3 (p > 0.05). 

The statistically significant difference between groups was found for the TAS, TOS, and copeptin levels. The TAS, TOS, and copeptin levels were found significantly higher in the epidural group than in the control and water groups (p = 0.036, p = 0.027, and p < 0.001 respectively). No significant difference was found between the water and control groups (p = 0.125). APGAR score showed negative correlation with cord blood TAS (r = 0.469, p < 0.001), TOS (r = 0.516, p < 0.001), and copeptin (r = 0.518, p < 0.001) levels with correlation analysis. The IL-1 and IL-6 levels were not different between the three groups (p = 0.234 and 0.114, respectively). 

The NICU need was significantly higher in the epidural group (p = 0.011). Need of NICU were 3.3%, 20%, 2.3% respectively in group 1, 2, and 3 (Table ). The admission etiology was respiratory problems for all the neonates and none of them stayed in NICU for more than a week. We only have the cord blood pH of the newborns who were sent to NICU. We did not detect a newborn who has cord blood pH < 7 and base deficit >12.0 mmol/L.

## 4. Discussion

Labor pain is an intense and unpleasant feeling that affects most women’s attitudes of birth in favor of C-sections. While selecting the ideal pain relief method for the laboring woman, maternal satisfaction, efficacy, maternal, and fetal safety are essential concerns [15]. We tried to make some comments on the oxidative, inflammatory, and neuroendocrine effects of these methods by evaluating the level of specific markers’ changes. 

Key results from the study demonstrated that women who had epidural analgesia had higher levels of Copeptin, TAS, and TOS levels than the water immersion and control group. In the epidural group, one and 5 min Apgar scores are lower, and NICU administrations are significantly much more than water immersion and control groups. No significant difference was found between water immersion and control groups.

Cluett et al. reported an updated systemic review to determine the maternal and fetal effects of water immersion during labor [16]. Fifteen trials between 1990 and 2015 were evaluated. Perineal trauma, breastfeeding, and duration of the labor were not different between immersion and no immersion [16]. Two comprehensive studies from the UK were undertaken to analyze the maternal and fetal effects of water immersion. No maternal or fetal adverse effects were found associated with water immersion [17,18]. Cluett et al. demonstrated that there is no increased risk of maternal-neonatal infection, neonatal death, NICU need, and maternal complications such as atonia and water embolism [19]. Compatibly to the recent literature, we found that the risk of perineal trauma, breastfeeding, and duration of the labor was not significantly different from water immersion (Table 1). We evaluated fetal wellbeing with Apgar score and NICU need, which were not incompatible with conventional birth (Table 1). Laboratory investigation of water immersion did not address increased oxidative stress, infection, or neuroendocrine deterioration (Table 2). Although studies advocate increased risk for infection and oxidative stress with water immersion, more comprehensive and evidence-based studies did not support this hypothesis [19–21]. Our study demonstrated that water immersion provides pain relief without altering oxytocin, which is essential for postpartum maternal adaptation to birth [5].

**Table 2:** The results of biochemical measurements.

	Group 1 (n = 29)	Group 2 (n = 30)	Group 3 (n = 29)	p-value
Copeptin (pg/mL)	66.44 (63.20–77.25)a,b	85.70 (77.90–95.62)a,c	59.72 (57.74–65.96)b,c	< 0.001†
Oxytosin (pg/mL)	21.55 (11.98–28.60)	19.44 (15.02–25.64)	18.91 (13.11–26.25)	0.887†
TAS(mmol/L)	1.24 (0.99–1.46)	2.26 (0.94–2.66)	1.4 (0.94–1.77)	0.036†
TOS (mmol/L)	4.26 (3.92–4.60)	5.77 (5.52–6.02)c	4.22 (3.99–4.45)c	0.027†
IL-1 (pg/mL)	4.66 (3.10–8.12)	4.27 (3.44–9.22)	4.34 (4.12–8.22)	0.234†
IL-6 (pg/mL)	8.12 (6.14–12.13)	8.23 (6.34–14.12)c	8.66 (3.22–14.53)c	0.114†

Epidural analgesia is one of the most popular medical pain relief methods during labor. Maternal and fetal outcomes are well studied in the literature, and no evidence was found to increase maternal and fetal adverse outcomes. According to the available data, epidural analgesia increases labor duration, which might impact fetal and maternal outcomes [15]. Gizzo et al. showed that the length of labor was longer with epidural analgesia without neonatal outcomes [22]. Our study found no significant difference in the period of active and overall labor duration (Table 2).

Similarly, Mousa et al. found no significant difference between epidural analgesia and control groups in terms of duration of labor, instrumental delivery, and Apgar scores [22]. Hasegawa et al. found that epidural analgesia is associated with lower pH and Apgar scores less than 7 with an increase in instrumental delivery [23]. Labor duration was also found longer than controls, and the difference in neonatal outcome was assumed to be associated with this prolongation [23]. In our study, we found that 1–5 min. Apgar scores were lower, and NICU needs, oxidative stress markers (TAS, TOS, and copeptin) were significantly higher in the epidural analgesia group without any difference regarding labor duration and instrumental delivery. 

The effect of epidural analgesia itself is the other point that needed to be evaluated to understand the mechanism underlying adverse fetal outcomes. Concentrations and type of anesthetic and the time anesthesia was performed are potentially useful parameters [15]. Sultan et al. evaluated the effect of low and high-dose local anesthetics on neonatal outcomes in their meta-analyze [24]. No significant difference was found for fetal heart rate anomalies, Apgar scores, and NICU need, although assisted vaginal deliveries were higher with high local anesthetic concentration [24]. The time of anesthesia (early or late administration) has no significant impact on fetal outcomes. However, cesarean rates tend to increase, according to Ismail et al. [25]. Our study performed all the epidural analgesia when the labor was active with cervical ripening more than 5 cm and intense regular contractions. A Cochrane review conducted in 2018 evaluated epidural analgesia regarding maternal and fetal effects [26]. According to the 40 trials results, epidural analgesia has no adverse impact on mother and fetus [26]. Distinctively, Gomez et al. found lower Apgar scores in the epidural group in their retrospective study of 2399 pregnant women [27]. In our study, the administration of epidural analgesia during labor was associated with lower Apgar scores at first and fifth minutes. However, the mean score in both groups was always >7 in the 3 groups. After re-evaluating the Apgar scores, which are lower than 7, the difference disappeared. 

In the present study, the odds of having a baby in need of NICU was significantly higher when epidural analgesia was performed in low-risk women. Similar results were found in Gomez et al.’s study, and they claimed that epidural analgesia might have adverse effects on neonatal outcomes [27]. In our research, we found a deteriorated oxidative status in the epidural group. This parameter could explain the increased need for NICU. The literature has inconsistent data regarding the effects of birth mode on oxidative stress [28,29]. Increasing stress and oxidant-antioxidant levels are a healthy physiological adaptation to the birth process, although it is known that pain management decreases the level of stress [30]. Literature seems almost to agree that analgesia reduces oxidative stress [31]. The limited number of patients included in the study is one of the weaknesses of our study. Although there is a statistically significant difference, there is no Apgar score of less than seven, which is clinically meaningful in the study group. However, some limitations for the study should be noted. Firstly, the sample size of the study would be larger to provide a more significant statistical evaluation. The other limitation is the lack of previous research studies on this topic. Thirdly, we did not observe the changes in fetal cord blood during the study period. The changes in fetal cord blood would reflect the acute effects of the labor method on newborns.

The breastfeeding rate of group 3 was found lower than the other groups in the first 24 h, Although the difference was not found significant. The breastfeeding status in the first 24 h should be analyzed from two different aspects: mother and newborn. The effect of the soluble medication which passes through the placenta during epidural analgesia might result in delayed breastfeeding with poor latching of the newborn onto the breast according to the study of Lind et al.[32]. Studies have shown that oxytocin which is essential for breastfeeding is found lower when the pain relief methods are used during the labor [32]These studies advocated that the use of pain relief would decrease the rate of early breastfeeding. If we look from the mothers’ aspect, labor is associated with enhanced catecholamine release especially in the second stage of labor. Pain and these catecholamines such as adrenalin result in maternal fear and anxiety presenting with autonomic responses such as tachycardia and elevated blood pressure [33]. Studies suggest that bad birth experience strongly affect the cessation and delaying of breastfeeding [34]. We think that the low rate of breastfeeding in group 3 is associated with the painful labor and tiredness of the mothers. We examined only the early breastfeeding of the women in this study. It should be investigated for general breastfeeding attitudes in further studies.

## 5. Conclusion

Our results suggest that epidural analgesia is associated with increased oxidant and antioxidant levels and more unsatisfactory neonatal outcomes than conventional birth and water birth. Further studies are needed to investigate the oxidative status and its potential effects on neonates with epidural analgesia.

## Funding

None. 

## Informed consent

Informed consent was obtained from all individual participants included in the study. The ethical committee of Zekai Tahir Burak Women’s Health and Research Hospital has approved the study protocol (38/2018*).
